# Microglia modulation with 1070-nm light attenuates Aβ burden and cognitive impairment in Alzheimer’s disease mouse model

**DOI:** 10.1038/s41377-021-00617-3

**Published:** 2021-09-08

**Authors:** Lechan Tao, Qi Liu, Fuli Zhang, Yuting Fu, Xi Zhu, Xiaofu Weng, Hongbin Han, Yong Huang, Yuanzhen Suo, Liang Chen, Xiaoling Gao, Xunbin Wei

**Affiliations:** 1 State Key Laboratory of Oncogenes and Related Genes, Shanghai Cancer Institute, Med-X Research Institute and School of Biomedical Engineering, Shanghai Jiao Tong University, Shanghai, 200030 China; 2 Institute of Medical Technology, Peking University Health Science Center, Beijing, 100191 China; 3 Department of Radiology, Peking University Third Hospital, Beijing, 100191 China; 4 Key Lab of Magnetic Resonance Imaging Device and Technique, Beijing, 100191 China; 5 Zhejiang Brainhealth Medical Technology Co., Ltd, Hangzhou, 314400 China; 6 Biomedical Pioneering Innovation Center, Peking University, Beijing, 100871 China; 7 School of Life Sciences, Peking University, Beijing, 100871 China; 8 Department of Neurosurgery, Huashan Hospital, Shanghai Medical College, Fudan University, Shanghai, 200040 China; 9 Tianqiao and Chrissy Chen Institute for Clinical Translational Research, Huashan Hospital, Shanghai, 200040 China; 10 Department of Pharmacology and Chemical Biology, State Key Laboratory of Oncogenes and Related Genes, Shanghai Universities Collaborative Innovation Center for Translational Medicine, Shanghai Jiao Tong University School of Medicine, Shanghai, 200025 China; 11 Biomedical Engineering Department, Peking University, Beijing, 100081 China; 12 Key Laboratory of Carcinogenesis and Translational Research (Ministry of Education/Beijing), Peking University Cancer Hospital & Institute, Beijing, 100142 China

**Keywords:** Biophotonics, Other photonics

## Abstract

Photobiomodulation, by utilizing low-power light in the visible and near-infrared spectra to trigger biological responses in cells and tissues, has been considered as a possible therapeutic strategy for Alzheimer’s disease (AD), while its specific mechanisms have remained elusive. Here, we demonstrate that cognitive and memory impairment in an AD mouse model can be ameliorated by 1070-nm light via reducing cerebral β-amyloid (Aβ) burden, the hallmark of AD. The glial cells, including microglia and astrocytes, play important roles in Aβ clearance. Our results show that 1070-nm light pulsed at 10 Hz triggers microglia rather than astrocyte responses in AD mice. The 1070-nm light-induced microglia responses with alteration in morphology and increased colocalization with Aβ are sufficient to reduce Aβ load in AD mice. Moreover, 1070-nm light pulsed at 10 Hz can reduce perivascular microglia and promote angiogenesis to further enhance Aβ clearance. Our study confirms the important roles of microglia and cerebral vessels in the use of 1070-nm light for the treatment of AD mice and provides a framework for developing a novel therapeutic approach for AD.

## Introduction

Photobiomodulation (PBM), also known as low-level light therapy, is a promising therapeutic approach and has been applied to various diseases as an alternative intervention^1–5^. PBM refers to the low-power light (1–500 mW) in the visible and near-infrared (NIR) spectra to trigger beneficial biological processes in cells and tissues, leading to physiological alterations^6^. Although the exact mechanisms of PBM are not yet elucidated, it is believed that NIR light can activate the chromophore, including cytochrome c oxidase and heat-gated ion channels^7–10^, as well as restore cellular function via multi-level mechanisms. PBM regulates the formation of reactive oxygen species and activates the transcription factors that can upregulate or downregulate expression levels of a large number of genes^3,11–13^. One of the most well-known transcription factors activated by PBM is nuclear factor kappa B, which regulates the expression of over 100 genes, including proteins with antioxidant, anti-apoptotic, pro-proliferative, and pro-migratory functions^7^. Therefore, PBM has been considered as a possible novel non-pharmacological and non-invasive therapeutic strategy for Alzheimer’s disease (AD)^14–19^, for which there are currently no effective therapeutic interventions^20^.

AD, the most common form of dementia, is associated with progressive impairments in memory and cognitive skills that affect daily activities throughout the disease course, ultimately leading to death^21^. One of the hallmark pathologies required for the diagnosis of AD is plaque deposits of the β-amyloid (Aβ) peptide, which can lead to neuronal loss, neuroinflammation, cognitive deficits^22^. PBM has been found to ameliorate cognitive and memory impairments in AD mouse models^3,15,16^ and AD patients^23,24^. There is compelling evidence suggested that PBM is capable of decreasing Aβ burden in the plasma^17^, cerebrospinal fluid^17^, and brain^19^ in AD models. A previous study from our group demonstrated the therapeutic effects of continuous-wave light with a wavelength of 1070 nm on memory abilities and Aβ burden in an AD mouse model^25^. Similarly, Grillo et al. demonstrated the beneficial effects of 1070-nm light on Aβ and phosphorylated tau protein levels in the brains of AD mice^15^. There is also evidence that the 1064-nm laser has beneficial neurocognitive effects in older adults^26^. Although a number of studies have shown the effects of continuous-wave NIR light on AD, the effects of NIR light with different pulse frequencies differ. Iaccarino et al. found that 40-Hz light flicker reduced Aβ and phosphorylated tau burden in the visual cortex of AD mice^27^. Hamblin’s group studied the differences in therapeutic effects between pulsed and continuous-wave 810-nm laser treatment. The results showed that 810-nm light pulsed at 10 Hz was the most effective in improving the neurological severity scores of mice with traumatic brain injury, compared with the 100-Hz pulsed laser and continuous-wave laser^28^. Furthermore, a number of studies demonstrated the beneficial effects of 10-Hz pulsed light on learning and memory impairments in certain diseases, such as sleep deprivation^29^, traumatic brain injury^30^, and mild to moderately severe dementia^23^.

Here, we studied the effects of 1070-nm light pulsed at 10 Hz and 40 Hz on APP_swe_/PS1_dE9_ (APP/PS1) mice, which is one of the most common animal models of AD^31^. We demonstrated that 1070-nm light could improve cognitive impairment via reducing the cerebral Aβ load. Engulfment by glial cells, such as microglia and astrocytes, plays a key role in the clearance of Aβ deposition^32–35^. Microglia and astrocytes, which tightly surround plaques of the brain in AD patients, can engulf and degrade different forms of Aβ^36–40^. Moreover, glial cells can protect the brain tissue from surrounding toxic Aβ species via the formation of glial capsules around Aβ deposits^41^. Therefore, we hypothesized that 1070-nm light could modulate glial cells to promote the clearance of Aβ burden in the brains of APP/PS1 mice, leading to improvements in memory and cognitive deficits. To test this, we studied the biological responses of microglia and astrocytes triggered by 1070-nm light and its effects on Aβ clearance and cognitive abilities. Our results showed that 1070-nm light pulsed at 10 Hz activated microglia rather than astrocytes to promote the degradation of Aβ. Moreover, the 10-Hz pulsed-light treatment reduced vessel-associated microglia and increased vessel density to further decrease Aβ load in APP/PS1 mice, ultimately leading to improvements in memory ability.

## Results

### 1070-nm light irradiation apparatus developed for APP/PS1 mice treatments

**Fig. 1:**
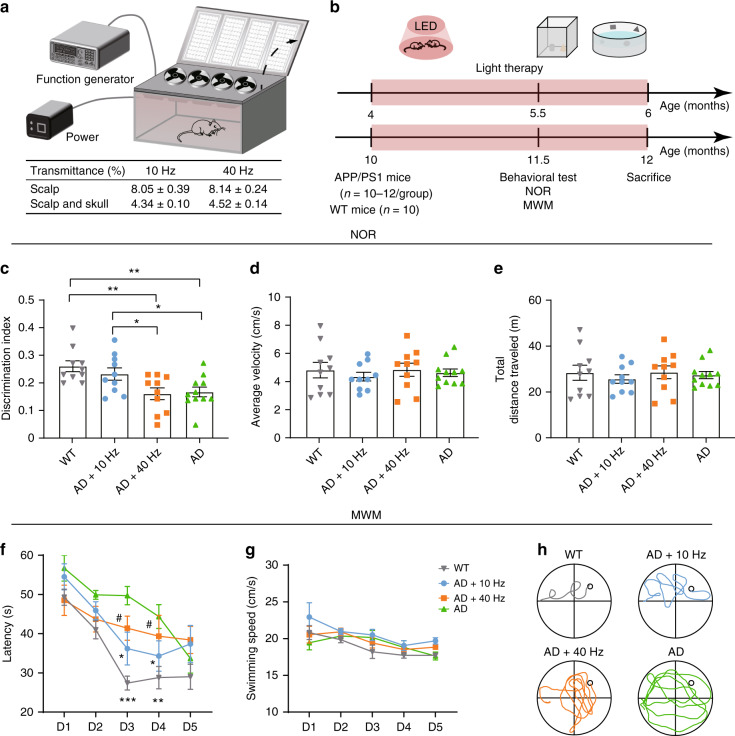
1070-nm light rescues cognitive impairment in APP/PS1 mice. **a** The 1070-nm light irradiation apparatus and the transmittances of 1070-nm light. **b** Experimental schematic of 1070-nm light irradiation and behavior tests. **c**–**e** The discrimination index (**c**), average velocity (**d**) and total distance traveled (**e**) in the four groups during the NOR test. Data in (**c**)–(**e**) are mean ± s.e.m., *n* = 10–11 per group, *p < 0.05, **p < 0.01. **f**–**h** Escape latency (**f**), swimming speed (**g**) and typical swimming path on day 4 (**h**) of mice during the MWM. Data in (**f**), (**g**) are mean ± s.e.m., *n* = 8–9 per group. Statistically significant differences between WT and AD + 40 Hz groups are indicated by the pound sign: # *P* < 0.05. Statistically significant differences between AD group and the rest of groups are indicated by asterisks: *p < 0.05, **p < 0.01, ***p < 0.001

A 1070-nm light irradiation apparatus was developed for non-invasive treatment of APP/PS1 double transgenic mice (Fig. 1a). The structure of the apparatus was described in the “Methods”. During irradiation, the mice were awake and free to explore the environment in the apparatus. The pulse frequency of light used was set to 10 Hz (duty cycle: 50%). We also tested the 1070-nm light pulsed at 40 Hz, with the same average power density and total fluence applied to compare the differences of therapeutic effects influenced only by the pulse frequency of light. Based on previous results^25^, the wavelength of the LED array was chosen as 1070 ± 50 nm, and the average power density was 25 mW/cm^2^. The transmittances of incident 1070-nm light were summarized in Fig. 1a. No differences were found in the transmittance between different light pulse frequencies. Meanwhile, these results showed that approximately 8% of the 1070-nm light incident power penetrated through the scalp and 4.3% of that power penetrated through the scalp and skull combined. Thus, the data demonstrated that irradiance at the brain was approximately 2–4 mW/cm^2^ when the incident irradiance was 25 mW/cm^2^.

### 1070-nm light rescues cognitive impairment in APP/PS1 mice

The experimental schematic was described as Fig. 1b. To test the effects of 1070-nm light on the APP/PS1 mice, mice were irradiated for 60 continuous days. During the final two weeks of irradiation, we conducted Morris water maze (MWM) and novel object recognition (NOR) tests to analyze the spatial learning and memory abilities of mice at 6 months old (6M) and 12 months old (12M). The discrimination index, which estimates the recognition ability of a novel object, was measured to determine short-term memory impairment. For 12M mice, the discrimination index decreased significantly in the APP/PS1 mice without irradiation (AD group) compared with the wild-type (WT) mice, and this was successfully rescued by administration of 1070-nm light pulsed at 10 Hz (Fig. 1c). The group of APP/PS1 mice with 10-Hz pulsed-light irradiation (AD+10 Hz) performed similarly to WT mice, while 40-Hz pulsed light failed to alleviate the short-term memory deficits observed in APP/PS1 mice (Fig. 1c). However, the average velocity and total distance traveled did not differ significantly (Fig. 1d, e) among groups during the NOR test, which indicated that the higher discrimination index was not due to general differences in activities.

We next applied the MWM to test the spatial learning and memory of mice after 1070-nm light irradiation. Escape latencies in all groups showed a progressive decline during training (Fig. 1f), and this decline was less significant as the training days progressed in the AD group and APP/PS1 mice with 40-Hz pulsed-light irradiation (AD+40 Hz) group. Mice in the AD group displayed longer escape latencies at days 3 and 4 compared with the WT mice, while the 1070-nm light pulsed at 10 Hz significantly decreased escape latency (Fig. 1f). Moreover, the results showed that mice in the WT and AD+10 Hz groups performed shorter swimming paths compared with the AD group, while there were no significant differences in average swimming speed among the four groups (Fig. 1g, h). In contrast, the results during the probe test showed no difference among groups (data not shown). During the spatial reversal test, the percentage of time spent in the southwestern (SW) quadrant was measured to determine the reversal learning impairment. Similarly, 1070-nm light pulsed at 10 Hz significantly raised the percentage of time in the SW quadrant compared with the AD group (Fig. S1a, b). However, in 6M mice, there were no significant differences in escape latency and time in the SW quadrant among groups (Fig. S1c–f), although the latency tended toward being shorter in the AD+10 Hz group, compared with the AD group (Fig. S1c, d). Overall, these results illustrated that 1070-nm light substantially ameliorated short-term memory and spatial learning abilities of APP/PS1 mice at 12M.

### 1070-nm light attenuates Aβ deposition in APP/PS1 mice

**Fig. 2:**
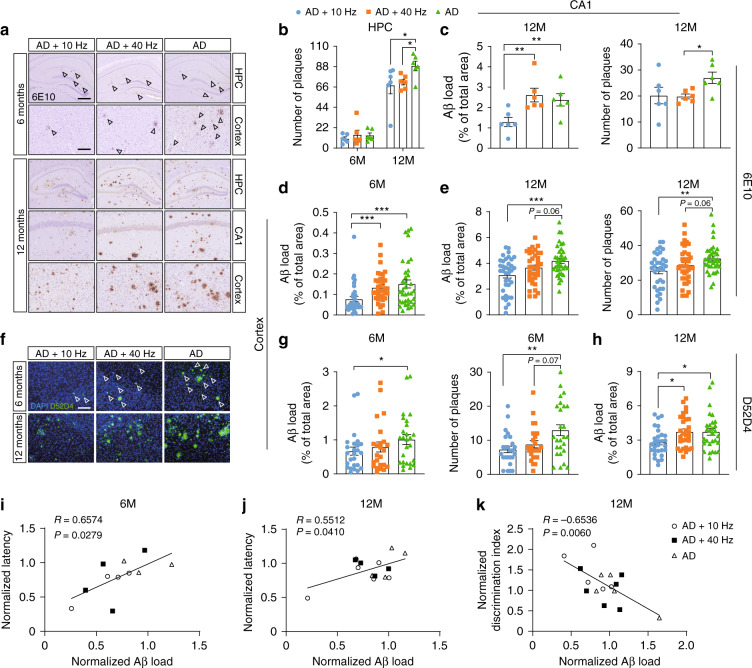
1070-nm light attenuates Aβ deposition in APP/PS1 mice. **a** Immunohistochemistry with anti-Aβ (6E10) antibody in the HPC, CA1 region and cortex of mice at 6M and 12M (scale bar, 400 μm in HPC, 100 μm in CA1 region and cortex). Arrowheads indicate the Aβ deposition. **b** The number of Aβ plaques in HPC of mice at 6M and 12M. **c**, **e** Area and number of Aβ plaques in CA1 region (**c**) and cortex (**e**) of mice at 12M. **d** Area of Aβ plaques in the cortex of mice at 6M. Data in (**b**)–(**e**) are stained with anti-Aβ (6E10) antibody. **f** Immunofluorescence with anti-Aβ (D54D2, green) antibody in the cortex of mice at 6M and 12M (scale bar, 100 μm). Arrowheads indicate the Aβ deposition. **g**, **h** The Aβ burden in the cortex of mice at 6M (**g**) and 12M (**h**). Data in (**g**), (**h**) are stained with anti-Aβ (D54D2) antibody. **i**, **j** The Pearson correlation coefficient analysis of Aβ load in the cortex of mice at 6M (**i**) and 12M (**j**) with the latency. **k** The Pearson correlation coefficient analysis between Aβ load in the cortex and the discrimination index in mice at 12M. Data in (**b**)–(**e**), (**g**), (**h**) are mean ± s.e.m., *n* = 6 per group for (**b**), (**c**), *n* = 30 and 25 fields of view from 6 and 5 mice per group for (**d**), (**e**), (**h**), and (**g**), *p < 0.05, **p < 0.01, ***p < 0.001. Data in (**i**)–(**k**) are from *n* = 3–6 per group

Aβ deposition in APP/PS1 mice induces neuronal dysfunction, ultimately leading to impairments in cognition. We thus explored whether PBM improves cognitive and memory deficits in APP/PS1 mice via reducing cerebral amyloid pathology. The hippocampus (HPC), which plays an important role in spatial memory and consolidation of short-term memory to long-term memory^42^, is the first brain region that suffers damage in AD^43^. Meanwhile, the cortex is associated with attention, memory, language and other cognitive activities. Therefore, we examined Aβ burden in the cortex and HPC of 6M and 12M APP/PS1 mice through immunostaining with anti-Aβ antibody (6E10) (Fig. 2a–e) and anti-Aβ antibody (D54D2) (Fig. 2f–h). Immunohistochemical results of anti-Aβ antibody staining (6E10) showed no significant differences in Aβ burden in the HPC among groups at 6M (Fig. 2a, b). In contrast, the total area of Aβ deposition in the cortex of 6M mice was reduced significantly in the AD+10 Hz group, compared with the AD+40 Hz (42.14%) and AD groups (48.96%), respectively (Fig. 2a, d). For mice at 12M, the results revealed significantly reduced plaque area size (CA1: 45.78%; Cortex: 26.34%) and number (HPC: 23.50%; CA1: 25.31%; Cortex: 21.70%) in the AD+10 Hz group, versus the AD group (Fig. 2a–c, e). Mice in the AD+40 Hz group also showed a decrease in the plaque number (HPC: 19.74%; CA1: 26.54%; Cortex: 11.98%), compared with the AD group (Fig. 2a–c, e).

Similarly, immunofluorescence with anti-Aβ antibody (D54D2) revealed a significant decrease in the plaque number by 43.53% and area by 33.74% in the cortex of 6M mice in the AD+10 Hz group compared with the AD group (Fig. 2f, g). A non-significant decrease was observed between the AD+40 Hz (area: 21.82%; number: 31.85%) and AD groups (Fig. 2f, g). However, the total area or number of Aβ deposition in the HPC showed no differences among groups (Fig. S2a, b). In mice at 12M, the results also showed that 1070-nm light pulsed at 10 Hz significantly decreased Aβ burden in the cortex by 21.23%, while no differences were found in the HPC or CA1 region, versus the AD group (Fig. 2f, h; Fig. S2a, c, d). Meanwhile, the number and area of plaques in the cortex, HPC or CA1 region did not differ significantly between the AD+40 Hz and AD groups (Fig. 2f, h; Fig. S2a, c, d).

To determine if there was a relationship between the performance of behavior tests and Aβ level, we also performed Pearson correlation coefficient analysis. The levels of Aβ in the cortex of mice both at 6M and 12M were positively correlated with latency (Fig. 2i, j). Meanwhile, there was a negative correlation between Aβ burden in the cortex of mice at 12M and the discrimination index during the NOR test (Fig. 2k). Taken together, these results demonstrated that 1070-nm light could reduce Aβ load in the cortex of APP/PS1 mice to improve its memory and cognitive abilities. Moreover, treating APP/PS1 mice with 1070-nm light in the earlier phase of AD tended to reduce more Aβ deposition.

### 1070-nm light reduces Aβ burden via eliciting microglia response in the cortex

**Fig. 3:**
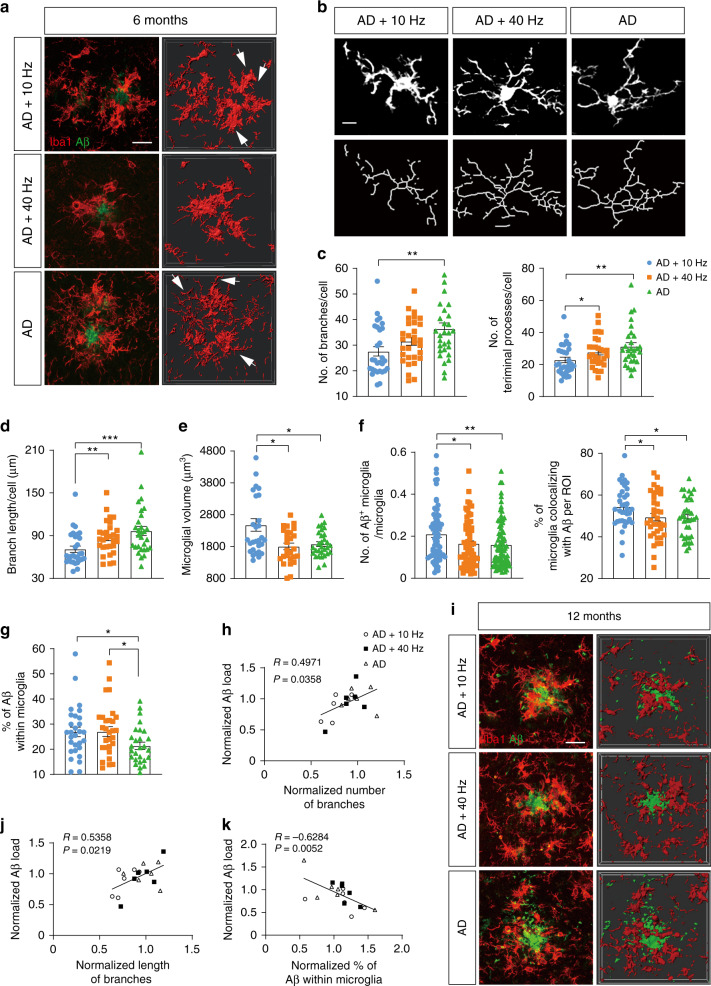
1070-nm light reduces Aβ burden via eliciting microglia response in the cortex. **a**, **i** Immunofluorescence with anti-Iba1 (red) and anti-Aβ (D54D2, green) antibodies in the cortex of APP/PS1 mice at 6M (**a**) and 12M (**i**) (scale bar, 20 μm). Arrowheads indicate the branches of microglia. **b** The topological skeletonized images of typical microglia from the three groups. Scale bar, 10 μm. **c**–**e** Number of branches and terminal processes per cell (**c**), branch length per cell (**d**), and microglial volume (**e**) in mice at 6M. **f**, **g** The colocalization between microglia and Aβ deposition in mice at 12M. **h**, **j** The Pearson correlation coefficient analysis of Aβ load with the number (**h**) and length (**j**) of branches in mice at 6M. **k** The Pearson correlation coefficient analysis between the Aβ load and percentage of Aβ within microglia in mice at 12M. Data in (**c**)–(**g**) are mean ± s.e.m., *n* = 30 fields of view from 6 mice per group for (**c**)–(**g**), *p < 0.05, **p < 0.01, ***p < 0.001. Data in (**h**), (**j**), (**k**) are from *n* = 5–6 per group

Engulfing and degrading Aβ protein via glial cell activity (e.g., astrocytes and microglia) is a main pathway for Aβ clearance in the AD^32–35^. Thus, the effects of 1070-nm light on astrocytes and microglia were tested to explore if light-activated microglia or astrocytes would promote Aβ clearance. Activated microglia were identified by a de-ramified phenotype which referred to retracted processes, enlarged cell bodies, and the high capacity for phagocytosis^44–46^. We thus analyzed the morphological alterations in microglia. For mice at 6M, we observed light-modified morphological features in microglia. Microglia were significantly reduced in number (24.13%) and length (26.59%) of branches after 10-Hz pulsed-light treatment, compared with the AD group (Fig. 3a–d). Likewise, the microglial volume was substantially increased by 31.99% in mice of the AD+10 Hz group, versus the AD group (Fig. 3e). Nevertheless, 40-Hz pulsed light failed to change the morphology of microglia in the cortex (Fig. 3a–e). For mice at 12M, the morphology of microglia did not differ among groups (Fig. S3a). However, the length and number of branches in mice at 12M decreased compared to 6M mice (Fig. 3c, d, Fig. S3a), which indicated that most microglia were activated in mice at 12M. There were also no differences in the ionized calcium-binding adapter molecule 1 (Iba1) burden among groups at 6M or 12M, further suggesting that 1070-nm light changed the morphology rather than the amount of microglia (Fig. S3b, c).

Activated microglia have a high capacity for engulfment^46,47^. The effects shown above regarding 1070-nm light and microglia activation led us to explore the colocalization between Aβ and microglia. Indeed, the AD+10 Hz group displayed an increase in the colocalization between microglia and Aβ in the cortex of mice at 12M, while no significant increase was observed in the AD+40 Hz group (Fig. 3f, g, i). For mice at 6M, there were no differences in the colocalization between Aβ plaques and microglia in the AD+10 Hz group, versus the AD mice (Fig. S3d). Furthermore, the results revealed that the number and length of branches were positively correlated with Aβ load in the cortex of 6M mice, respectively (Fig. 3h, j). There was also a negative correlation between the percentage of Aβ within microglia and Aβ load in the cortex of 12M mice (Fig. 3k).

In addition to microglia, astrocytes also uptake and degrade different forms of Aβ. We thus assessed the effects of 1070-nm light on astrocytes in AD mice. For mice at 6M, glial fibrillary acidic protein (GFAP)-positive areas in the cortex were reduced by 23.99% in the AD+40 Hz group, while 10-Hz pulsed light failed to reduce GFAP-positive cells, compared with the AD group (Fig. S4a, b). Mice in the AD+40 Hz group showed a 16.31% increase in the colocalization between astrocytes and Aβ (Fig. S4a, b). However, no significant differences in GFAP-positive areas or colocalization of astrocytes with Aβ in the cortex were found in 12M mice (Fig. S4a, c). Moreover, the correlation analysis showed that the 1070-nm light-induced alterations in mice at 6M were not correlated with Aβ load or latency (Fig. S4d-f). Overall, these results indicated that 1070-nm light-activated microglia rather than astrocytes to promote the degradation of Aβ.

### 1070-nm light decreases M1-like microglia surrounding the vessels in the cortex

**Fig. 4:**
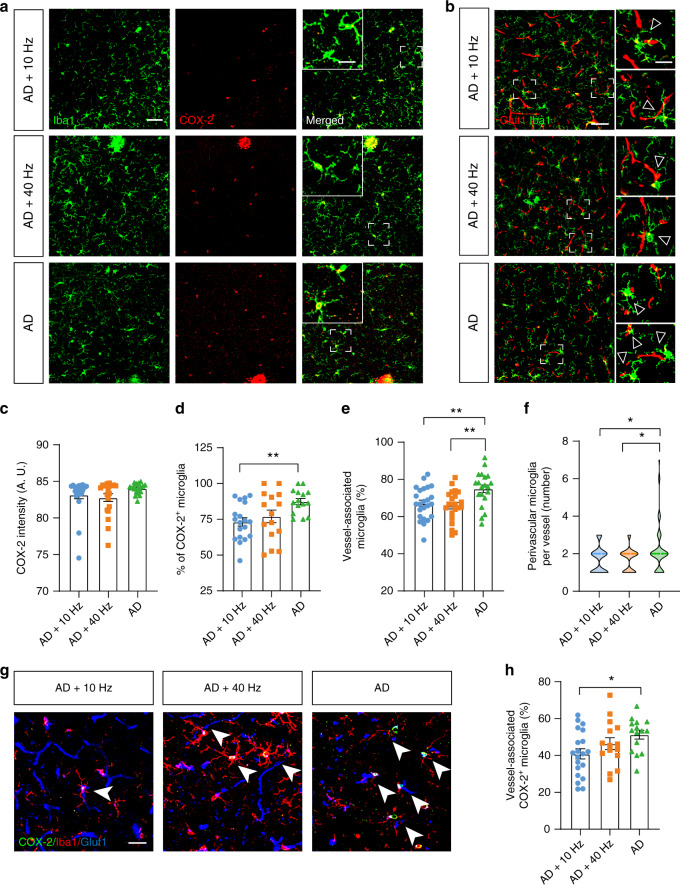
1070-nm light decreases M1-like microglia surrounding the vessels in the cortex of APP/PS1 mice at 6M. **a** Immunofluorescence with anti-Iba1 (green) and anti-COX-2 (red) antibodies in the cortex of APP/PS1 mice at 6M (scale bar, 50 μm). The magnified images show the typical microglia expressing COX-2 (scale bar, 20 μm). **b** Immunofluorescence with anti-Iba1 (green) and anti-Glut1 (red) antibodies in the cortex of mice at 6M (scale bar, 50 μm). Arrowheads and the magnified images show the typical blood vessels with associated microglia (scale bar, 20 μm). **c**, **d** COX-2 fluorescence intensity (**c**) and the percentage of COX-2 positive microglia (**d**) in mice at 6M. **e**, **f** The percentage of vessel-associated microglia (**e**) and the number of perivascular microglia per vessel (**f**) in mice at 6M. **g** Immunofluorescence with anti-Iba1 (red), anti-COX-2 (green), and anti-Glut1 (blue) antibodies in the cortex of mice at 6M (scale bar, 25 μm). Arrowheads indicate contact between COX-2^+^ microglia and blood vessels. **h** The percentage of COX-2^+^ microglia surrounding vessels in the cortex of mice at 6M. Data in (**c**)–(**f**) and (**h**) are mean ± s.e.m., *n* = 15–20 fields of view from 3–4 mice per group for (**c**)–(**e**) and (**h**), *n* = 60–63 vessels from 4 mice per group for (**f**), *p < 0.05, **p < 0.01

Activated microglia have two different polarization states. M1-like microglia, which produce pro-inflammatory factors, are increased in AD patients. It leads to oxidative stress and cognitive deficits^48^. In contrast, M2-like microglia exert a neuroprotective effect and induce tissue repair^49,50^. To assess whether 1070-nm light would change M1/M2 polarization to decrease the impairment induced by M1-like microglia, we analyzed M1 and M2 markers, respectively. Results showed that the fluorescence intensity of the M1 marker, Gyclooxygenase-2 (COX-2), had no differences among the three groups (Fig. 4a, c). In contrast, the percentage of COX-2^+^ microglia was decreased by 16.30% in the AD+10 Hz group at 6M, compared with the AD group (Fig. 4a, d). For mice at 12M, 1070-nm light pulsed at 10 Hz significantly reduced the fluorescence intensity of COX-2 in the cortex, compared with the AD (16.58%) and AD+40 Hz groups (24.05%), indicating that M1-like microglia were reduced after 10-Hz pulsed-light irradiation (Fig. S5). In contrast, few microglia with M2 marker CD163 were found among the three groups (Fig. S6), indicating that there were not many M2-like microglia in the AD mice.

Perivascular microglia, especially perivascular M1-like microglia, stimulate endothelial cells and pericytes of the blood-brain barrier, as well as decrease tight junction proteins, ultimately leading to cerebrovascular dysfunction in AD^51^. We thus explored if 1070-nm light would reduce perivascular M1-like microglia to increase vessel density. To test this, we analyzed the perivascular microglia. The results showed that there was a decrease in the number of perivascular microglia in the 1070-nm light groups at 6M (Fig. 4b, e, f). Moreover, there was also a decrease in M1-like microglia surrounding vessels in the AD+10 Hz group at 6M (20.35%), while 40-Hz pulsed light failed to reduce the M1 phenotype in perivascular microglia (Fig. 4g, h). Altogether, these results indicated that 1070-nm light pulsed at 10 Hz could reduce vessel-associated microglia and perivascular M1-like microglia.

### 1070-nm light decreases Aβ deposition via increasing cerebral vessel density

**Fig. 5:**
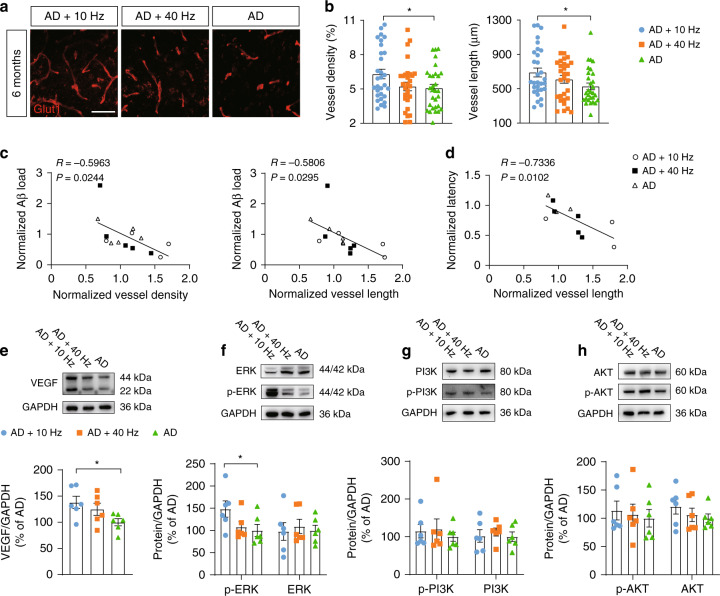
1070-nm light decreases Aβ deposition via increasing cerebral vessel density. **a** Immunofluorescence with anti-Glut1 antibody in the cortex of mice at 6M (scale bar, 50 μm). **b** Vessel density and length in the cortex of mice at 6M. **c**, **d** The Pearson correlation coefficient analysis of vessel density and length with Aβ load (**c**) and latency (**d**) in mice at 6M. **e**–**h** Western blot analysis for VEGF (**e**), ERK, p-ERK (**f**), PI3K, p-PI3K (**g**), AKT, p-AKT (**h**) and GAPDH in the cortex of mice at 6M. Data in (**b**), (**e**)–(**h**) are mean ± s.e.m., *n* = 30 fields of view from 6 mice per group for (**b**), *n* = 6 per group for (**e**)–(**h**), *p < 0.05. Data in (**c**), (**d**) are from *n* = 3–5 per group

The results in the perivascular microglia led us to investigate if 1070-nm light could rescue the decreased vessel density in APP/PS1 mice. For mice at 6M, increased vessel density (24.26%) and length (30.74%) in the cortex were found in the AD+10 Hz group, versus the AD group (Fig. 5a, b). In addition, there was a slight, non-significant increase in vessel length (15.56%) in the cortex of the AD+40 Hz group (Fig. 5a, b). Furthermore, Pearson correlation coefficient analysis displayed a strong correlation of 1070-nm light-induced alterations of vessel with Aβ load and latency in MWM (Fig. 5c, d), which indicated that 1070-nm light could also promote the clearance of Aβ deposition in mice at 6M via amelioration of cerebral vessel impairment.

A previous study showed that high vessel endothelium growth factor (VEGF) levels in the brains of patients were positively correlated with good performance in cognitive tests^52^. We thus analyzed the levels of VEGF in the cortex of mice at 6M. The results showed that VEGF levels were increased by 38.12% in the AD+10 Hz group, versus the AD group (Fig. 5e). The extracellular signal-regulated kinase (ERK) pathway, which is a key signaling pathway that regulates a wide variety of cellular processes, can mediate the levels of VEGF^53^. Therefore, we examined the levels of phosphorylated ERK (p-ERK) and ERK. A significant increase in p-ERK levels was found in the AD+10 Hz (48.19%) versus the AD group, while the ERK level did not differ significantly among groups (Fig. 5f). The phosphoinositide 3-kinase (PI3K)/protein kinase B (AKT) pathway, which is an important intracellular signaling pathway in regulating the cell cycle, also mediates the levels of VEGF^54^. However, the results showed no differences in the levels of phosphorylated PI3K (p-PI3K), PI3K, phosphorylated AKT (p-AKT) or AKT among groups at 6M (Fig. 5g, h). Overall, 1070-nm light could modulate the VEGF levels to increase cerebral vessel density in the cortex of APP/PS1 mice at early pathological stage. Moreover, increased vessel density in the 1070-nm light group could promote the clearance of Aβ.

## Discussion

In the present study, the main findings have demonstrated the therapeutic effects of 1070-nm light on the memory and cognition impairments, as well as on Aβ clearance in APP/PS1 mice. Our results show that 1070-nm light pulsed at 10 Hz triggers microglia responses with alteration in morphology and increase in its colocalization with Aβ, instead of triggering astrocyte responses. The responses of microglia to 1070-nm light are negatively correlated with the level of Aβ, suggesting that 1070-nm light pulsed at 10 Hz can reduce the Aβ burden via eliciting microglia activation and recruiting microglia to Aβ deposition. The perivascular microglia, especially M1-like microglia, decrease after 10-Hz pulsed-light treatment, while an increase in cerebral vessel density is found in AD+10 Hz group. Moreover, vessel density is positively correlated with clearance of Aβ deposition, suggesting that 1070-nm light pulsed at 10 Hz can also reduce Aβ burden via increasing cerebral vessel density. Overall, our findings suggest that 1070-nm light pulsed at 10 Hz can reduce cerebral Aβ levels and thus improve memory and cognition abilities through activation of microglia and promotion of angiogenesis. Therefore, 1070-nm light may be a practical and promising novel therapeutic strategy for treating AD.

Iaccarino et al. demonstrated that 40-Hz visual stimulation increased the power of gamma oscillation to reduce Aβ burden in the visual cortex via activation of microglia and changes in the morphology of microglia in 5xFAD mice^27^. Moreover, they found that gamma entrainment using sensory stimulus (GENUS) could improve the cognitive and memory impairments in 5xFAD mice and reduce Aβ load in the HPC^55^. Here, our results indicated that 1070-nm light pulsed at 10 Hz was more effective in treating AD mice compared with the 40-Hz pulsed light. The 10-Hz pulsed light used in this study can induce alterations in the widespread cortex, while the effects of gamma visual stimulation focus on the visual cortex. Meanwhile, the 10-Hz pulsed light used here is invisible so that it cannot trigger GENUS (responding to visible light), further suggesting that the mechanisms underlying the beneficial effects of 10-Hz pulsed-light stimulation and GENUS are different. One explanation as to why 1070-nm light pulsed at 10 Hz is more effective for treating AD is that its period (100 ms) is similar to the duration of some biological activities involved in the light stimulation^28^. Transient receptor potential (TRP) channels, the calcium ion channels, is reported to serve as photoreceptors and are responsible for some of the mechanisms of PBM^56–59^. Moreover, the half-life of the burst length of TRPV1 with activation of 0.25-μM capsaicin is 94.5 ± 30 ms, suggesting that 10-Hz pulsed-light stimulation may activate related ion channels to trigger a series of biological responses. However, more studies are necessary to explore mechanisms of the pulse frequency-dependent effects of 1070-nm light treatment on AD.

Previous studies have shown a reduction of Aβ burden in AD mice after 1070-nm light treatment^17,19^. However, few studies have focused on its effects at different stages of pathological progression. In this study, our results showed a trend that irradiating mice in the early pathological stage of AD was more effective to treat AD than those in the late stage (Fig. 2d, e, g, h). Das et al. found that treating APP Tg2576 with γ-secretase inhibitor during the pre-deposition “seeding” phase (4–7M) had the most significant efficacy on the reduction in Aβ burden. In contrast, treatment during the exponential phase of deposition (7–10M or 12–15M) showed progressively decreasing efficacy of treatment^60^. Our findings further suggested the more significant impact of 1070-nm light treatment occurred during the early stage of AD compared with the later stage. The 1070-nm light might reduce more Aβ plaques at the pre-deposition phase by attenuating the seeding of Aβ, which was critical to the development of Aβ deposition. In contrast, the effects of 1070-nm light on Aβ load in the later stage were not sufficient, considering the rapid growth rate of Aβ plaques. Therefore, 1070-nm light had less significant efficacy on the Aβ clearance in the late stage of AD.

The morphological alteration of microglia, which indicates microglia activation, appears at the early stage of AD^61^. Activated microglia can reduce the Aβ burden at the early stage of AD via increasing its phagocytosis and degradation^62,63^. Similarly, we have found that 1070-nm light promotes the activation of microglia to reduce the Aβ burden at the early stage of AD (Fig. 3a-e, h, j). Although a large number of microglia are activated with the pathological progression^61,64^, microglia show inefficient phagocytic and clearing ability of Aβ^65,66^ at the late stage of AD. Our results indicate that 1070-nm light promotes the phagocytic ability of microglia at the late stage of AD (Fig. 3f, g, i, k). Therefore, 1070-nm light is likely to enhance the innate responses of microglia to Aβ clearance which change with the pathological progression of AD.

One particular study demonstrated that 800-nm laser increased the M2-like microglia and decreased M1-like microglia, resulting in improvements in spinal cord injury^67^. Leden et al. also showed that 808-nm light induced a dose-dependent alteration in M1/M2 polarization and promoted neurite growth^68^. Similarly, our results displayed that M1-like microglia declined after 10-Hz pulsed-light irradiation. Microglia were reported to localize to perivascular Aβ depositions^69^. These plaque-associated microglia express inflammatory factors, such as interleukin-1β and tumor necrosis factor-α and mostly exhibit an M1-like phenotype^70^. Similarly, we found that increased perivascular microglia in AD mice and they were reduced after 1070-nm light treatment. The M1-like microglia around vessels were also decreased by 1070-nm light pulsed at 10 Hz. These perivascular microglia, especially perivascular M1-like microglia, promoted cerebrovascular dysfunction, leading to the deficiency in Aβ clearance and cerebral metabolism. Indeed, we observed an increase in cerebral vessel density after 10-Hz pulsed-light irradiation, suggesting that 1070-nm light pulsed at 10 Hz may promote vessel density via microglia modulation. In addition, our results demonstrated that 1070-nm light pulsed at 10 Hz increased VEGF levels and activated VEGF-related cellular pathways. A number of studies also found the upregulation of VEGF levels via the ERK pathway after light irradiation^4,71,72^. Therefore, this indicated another possible mechanism underlying the increase of vessel density, which was that the 1070-nm light pulsed at 10 Hz upregulated VEGF levels to promote angiogenesis in the cortex.

In conclusion, our results demonstrate the effects of 1070-nm light on microglia modulation and cerebral vessels during different phases of AD and provide valuable insight into the mechanisms of 1070-nm light treatment to AD. This is beneficial for the exploration of optimal parameters when administering PBM as well as the development of a promising and novel therapeutic approach for AD.

## Materials and methods

### Animals

Female APP_swe_/PS1_dE9_ (APP/PS1) double transgenic mice and age-matched wild-type (WT) littermates were utilized to explore the effects after 1070-nm light treatment. APP/PS1 mice overexpressing the hAPP695swe (APP695swe) and mutant human presenilin 1(PS1-dE9) on the C57BL/6 background were obtained from Nanjing Biomedical Research Institute of Nanjing University. To explore the differences of effects after 1070-nm light treatment at different onset stages, 4M and 10M mice received 60-day treatment, respectively. All mice with different ages were divided into four groups (*n* = 10–12 per group): one treatment group with 10-Hz pulsed-light irradiation (AD+10 Hz), one treatment group with 40-Hz pulsed-light irradiation (AD+40 Hz), one sham treatment group (AD), and one negative control group (WT). The AD group and WT group represented APP/PS1 mice with non-irradiated and normal mice, respectively. All mice were housed under a 12/12-h light-dark cycle with food and water ad libitum. Animal care and experimental protocols were approved by the Shanghai Jiao Tong University Ethical Committee of Animal Experiments.

In the last 15 days of irradiation treatment, we tested the memory and spatial learning abilities of all mice via the NOR and MWM tests (Fig. 1b). After behavioral tests, mice were humanely sacrificed. The brains were quickly dissected and stored at −80 °C until ready for further analysis.

### Apparatus and treatment

The apparatus consisted of a chamber and a light-emitting diode (LED) array as the lid. The average power of the LED array was 900 mW, and the average power density and the total fluence were 25 mW/cm^2^ and 4.5 J/cm^2^, respectively. The light pulse frequencies were set to 10 Hz and 40 Hz (duty cycle: 50%). The cooling fans were utilized to reduce the influence of thermal effects induced by the LED array.

For 1070-nm light treatment, mice in the AD+10 Hz and AD+40 Hz groups were placed in the 1070-nm light device and received irradiation of 6 min per day at 7 P.M. for 60 consecutive days, with the wavelength at 1070 ± 50 nm. During treatments, mice can ambulate, explore, and rest. Mice in the AD and WT groups underwent the same procedures as the treatment groups, except that the 1070-nm light device remained off.

### Behavioral tests

#### Novel object recognition test

The NOR test, which relies on a rodent’s natural proclivity for exploring novelty, is a common method for examining cognition, particularly recognition memory^73^. We performed the NOR test on day 46 of 1070-nm light treatment to measure the recognition memory of mice. During the habituation session, mice were placed into the empty open field and allowed to freely explore for 5 min. After a 24 h period, two identical non-toxic objects were placed in opposite and symmetrical corners of the arena; each mouse was released into the open field and allowed free exploration for a 10-min period. One of the previously explored objects was replaced 6 h later by a novel object. The mice were returned to explore the open field for an additional 10 min to test preference for a novel object. Object preference was analyzed by a discrimination index, calculated as follows:

DI = (*T*_novel_ − *T*_familiar_)/(*T*_novel_ + *T*_familiar_), where *T*_novel_ and *T*_familiar_ indicate the exploration time during testing for the novel and familiar objects, respectively.

#### Morris water maze test

The MWM, a widely accepted method for analyzing spatial learning and memory abilities^74^, was performed. Briefly, the apparatus consisted of a white plastic pool (120 cm in diameter and 50 cm in depth) filled with water (22 ± 1 °C), and a transparent escape platform (10 cm, square) located 0.5 cm to 1 cm below the water surface. The white edible pigment was added to the water to make the platform invisible during tests. The pool was divided into four quadrants. Mice were introduced to different quadrants randomly to learn how to find the hidden platform within 60 s by four extra maze cues placed asymmetrically as spatial references. If the mice failed to find the platform within 60 s, they were placed onto the platform and allowed to stay on top for 15 s to acclimatize with the surroundings. During the spatial test, all mice were trained for a period of 5 days with four trials per day. At the end of learning, a probe trial was conducted 24 h later with a hidden platform removed. After the spatial test, the mice were administered another set of four trials per day for 5 days and a 1-day probe trial to assess reversal learning. The mice were trained to relocate the platform in the opposite quadrant for 5 additional days, following by the reversal probe trial 24 h later, with the hidden platform removed again. All experiments were recorded by a computerized tracking system, which calculated distances moved and latencies required for reaching the platform.

### Tissue preparation

After the behavior test, mice were transcardially perfused with cold phosphate-buffered saline (PBS) under deep anesthesia. Brains were removed and separated into the two hemispheres, which were either fixed in 4% paraformaldehyde (PFA) or fresh-frozen on ice. The cortex and HPC were dissected out from fresh hemispheres and homogenized in RIPA (50 mM Tris HCl, pH = 8.0, 150 mM NaCl, 1% NP-40, 0.5% sodium deoxycholate, 0.1% SDS) buffer which contained protease and phosphatase inhibitor. The homogenates were incubated on ice for 20 min and centrifuged at 18,000 × *g* for 20 min before supernatants were collected and stored at −80 °C. Fixed hemispheres were kept in 4% PFA at 4 °C for 24 h and transferred to 30% sucrose solutions in PBS for 48 h. They were then subsequently snap-frozen in isopentane and stored at −80 °C until processing.

### Immunofluorescence

The brains were coronally sectioned on a freezing microtome at 20 µm thickness. For immunofluorescence, sections were blocked in 5% bovine serum albumin with 0.3% Triton X-100 for 60 min followed by overnight incubation with the primary antibody at 4 °C. The primary antibodies were anti-Aβ (1:100, 8243, Cell Signaling Technology), anti-Aβ antibody (1:400, 803004, Biolegend), anti-Iba1 antibody (1:1000, 019-1947, Wako), anti-GFAP antibody (1:1000, MAB360, EMD Millipore), anti-Glut1 antibody (1:100, ab40084, Abcam), anti-COX-2 antibody (1:200, 12282, Cell Signaling Technology), and anti-CD163 antibody (1:200, ab182422, Abcam). Thereafter, sections were incubated with goat anti-rabbit IgG Alexa Fluor 488 antibody (1:500, ab150077, Abcam), donkey anti-rabbit IgG Alexa Fluor 555 antibody (1:500, ab150074, Abcam), donkey anti-mouse IgG Alexa Fluor 488 antibody (1:500, ab150105, Abcam) or Goat anti-mouse IgG HRP-conjugated antibody (1:500, 405306, Biolegend) for 1 h at room temperature. The sections were mounted through a mounting medium with DAPI (H-1200, Vector laboratories). Images were acquired by a confocal microscope (SP8, Leica) with a 40X objective at identical settings for all conditions.

### Histological analysis

Images were analyzed using ImageJ software (National Institutes of Health, USA). For CA1 imaging, one field of view (293 × 293 µm) per mouse was measured. The cortex was analyzed by measuring the alteration in random 4–6 fields of view (293 × 293 µm) in the cortex per mouse. The morphology of microglia was measured by ImageJ. Meanwhile, the Coloc2 plugin was used to quantify the colocalization of Aβ with microglia and astrocytes. Three-dimensional reconstruction and volume of microglia were analyzed using IMARIS software (Bitplane). The COX-2 positive microglia per field of view were counted by an experimenter blind to treatment groups. For vessel-associated microglia, three randomly selected vessels per field of view in the cortex were analyzed. The perivascular microglia were quantified using the Leica Microscope Imaging Software by counting and comparing cells associated and not associated with blood vessels.

### Western blot

For Western blotting, the total protein of each sample was measured by the BCA protein assay kit (Thermo Scientific, Rockford, USA). The protein from each sample was separated by 4–12% NuPAGE (180-8018H, Tanon). After transfer of proteins to polyvinylidene fluoride membrane (Millipore, Billerica, USA), the membrane was blocked with 5% skim milk for 1.5 h at room temperature and incubated for 2 h at room temperature with the primary antibodies: anti-VEGF antibody (1:1000, 19003-1-AP, Proteintech), anti-PI3K antibody (1:1000, 4257, Cell Signaling Technology), anti-p-PI3K antibody (1:1000, 4228, Cell Signaling Technology), anti-AKT antibody (1:1000, 4691, Cell Signaling Technology), anti-p-AKT antibody (1:2000, 4060 T, Cell Signaling Technology), anti-ERK antibody (1:1000, 4695, Cell Signaling Technology), anti-p-ERK antibody (1:2000, 4370, Cell Signaling Technology), and anti-GAPDH antibody (1:10000, 60004-1-Ig, Proteintech). Thereafter, the membrane was incubated with HRP-conjugated secondary antibody at room temperature for 1 h and treated with enhanced chemiluminescent reagent kit (Thermo Scientific, Rockford, USA). The bands were scanned and digitalized. The density of each band was quantified using ImageJ and normalized to the values of GAPDH.

### Statistical analysis

Data were shown as mean ± standard error of the mean. The statistical analysis of the data was analyzed using GraphPad Prism 8.0. Comparisons for normally distributed data with two groups were analyzed by two-tailed unpaired t-tests. Comparisons for normally distributed data with three or more groups were tested by one-way ANOVA. Mann–Whitney tests were applied to determine statistical significances of data with the non-normally distribution. Statistical differences for all tests were considered significant at *p* < 0.05.

## Supplementary information

Supplementary materials
