# Supplementary material for: Microglia modulation with 1070-nm light attenuates Aβ burden and cognitive impairment in Alzheimer’s disease mouse model
Source: Light Sci Appl. 2021 Sep 8;10:179. doi: 10.1038/s41377-021-00617-3 (PMC8423759; doi:10.1038/s41377-021-00617-3)
Supplement: Supplementary file 1 — Supplementary materials [file 41377_2021_617_MOESM1_ESM.docx]

*Supplementary Information for:*

Microglia modulation with 1070-nm light attenuates Aβ burden and cognitive impairment in Alzheimer’s disease mouse model

Lechan Tao^1^, Qi Liu^1^, Fuli Zhang^1^, Yuting Fu^1^, Xi Zhu^1^, Xiaofu Weng^1^, Hongbin Han^2, 3, 4^, Yong Huang^5^, Yuanzhen Suo^6, 7^, Liang Chen^8, 9*^, Xiaoling Gao^10*^ and Xunbin Wei^1, 2, 11, 12*^

^1^ State Key Laboratory of Oncogenes and Related Genes, Shanghai Cancer Institute, Med-X Research Institute and School of Biomedical Engineering, Shanghai Jiao Tong University, Shanghai 200030, China;

^2^ Institute of Medical Technology, Peking University Health Science Center, Beijing, 100191, China;

^3^ Department of Radiology, Peking University Third Hospital, Beijing, 100191, China;

^4^ Key Lab of Magnetic Resonance Imaging Device and Technique, Beijing, 100191, China;

^5^ ZheJiang Brainhealth Medical Technology Company, Zhejiang, 314400, China;

^6^ Biomedical Pioneering Innovation Center, Peking University, Beijing, 100871, China;

^7^ School of Life Sciences, Peking University, Beijing, 100871, China;

^8^ Department of Neurosurgery, Huashan Hospital, Shanghai Medical College, Fudan University, Shanghai, 200040, China;

^9^ Tianqiao and Chrissy Chen Institute for Clinical Translational Research, Huashan Hospital, Shanghai, 200040, China;

^10^ Department of Pharmacology and Chemical Biology, State Key Laboratory of Oncogenes and Related Genes, Shanghai Universities Collaborative Innovation Center for Translational Medicine, Shanghai Jiao Tong University School of Medicine, Shanghai, 200025, China;

^11^ Biomedical Engineering Department, Peking University, Beijing, 100081, China;

^12^ Key Laboratory of Carcinogenesis and Translational Research (Ministry of Education/Beijing), Peking University Cancer Hospital & Institute, Beijing, 100142, China;

Correspondence: Prof. Liang Chen (hschenliang@fudan.edu.cn) or Prof. Xiaoling Gao (shellygao1@sjtu.edu.cn) or Prof. Xunbin Wei (xwei01@sjtu.edu.cn)

*
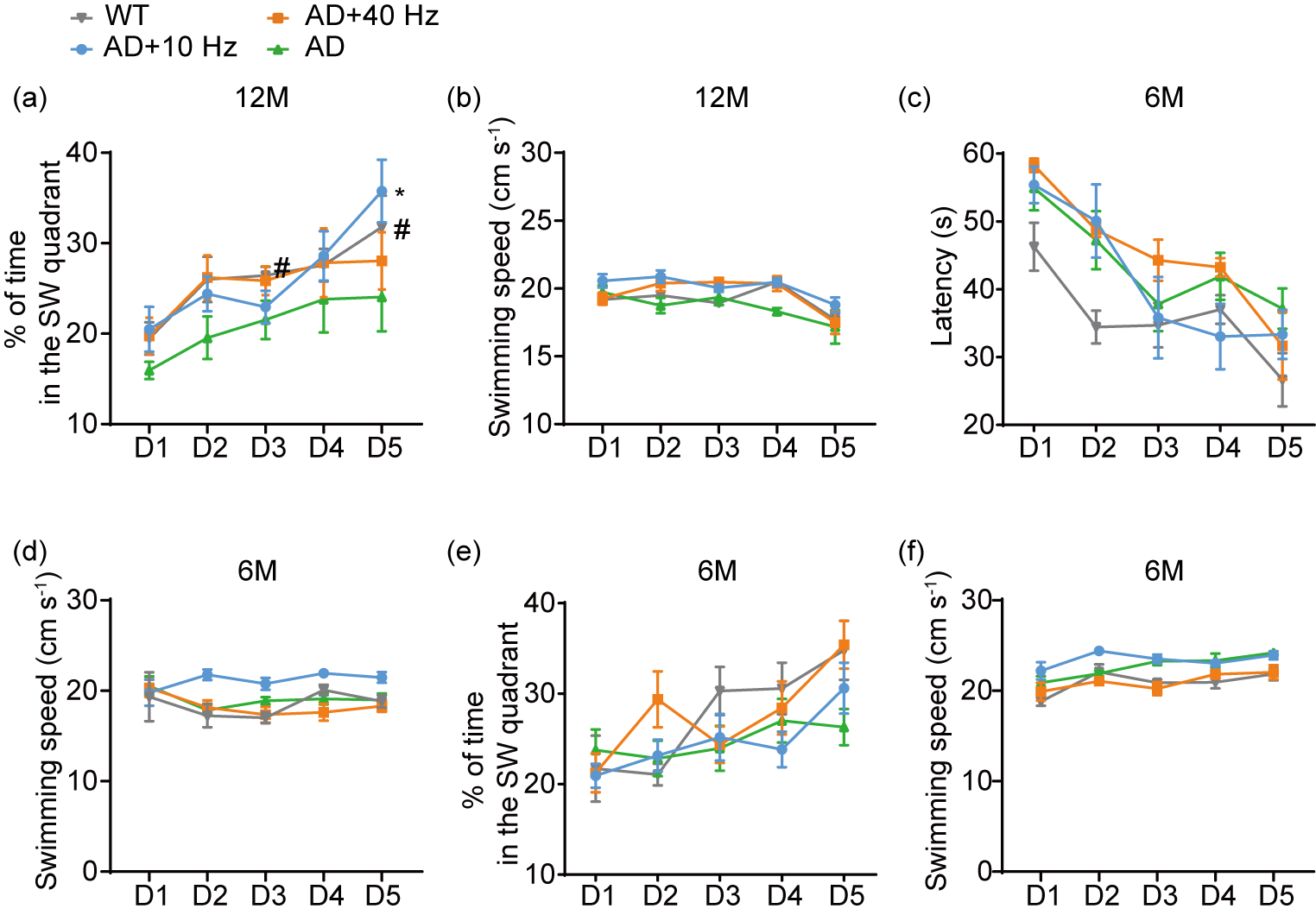
*

**Fig. S1.** The performance of APP/PS1 mice at 6M and 12M during MWM. (a), (b) The percent time in the SW quadrant (a) and swimming speed (b) of mice at 12M during reversal test. (c) Escape latency of mice at 6M during the spatial test. (d), (f) The swimming speed of mice at 6M during the spatial test (d) and reversal test (f). (e) The percent time in the SW quadrant of mice at 6M during reversal test. Date in (a)-(f) are mean ± s.e.m. Data in (a), (b) are from n = 8-9 per group. Data in (c)-(f) are from n = 7-8 per group. Statistically significant differences between WT and AD groups are indicated by the pound sign: # P < 0.05. Statistically significant differences between AD+10 Hz and AD groups are indicated by asterisks: * p < 0.05.


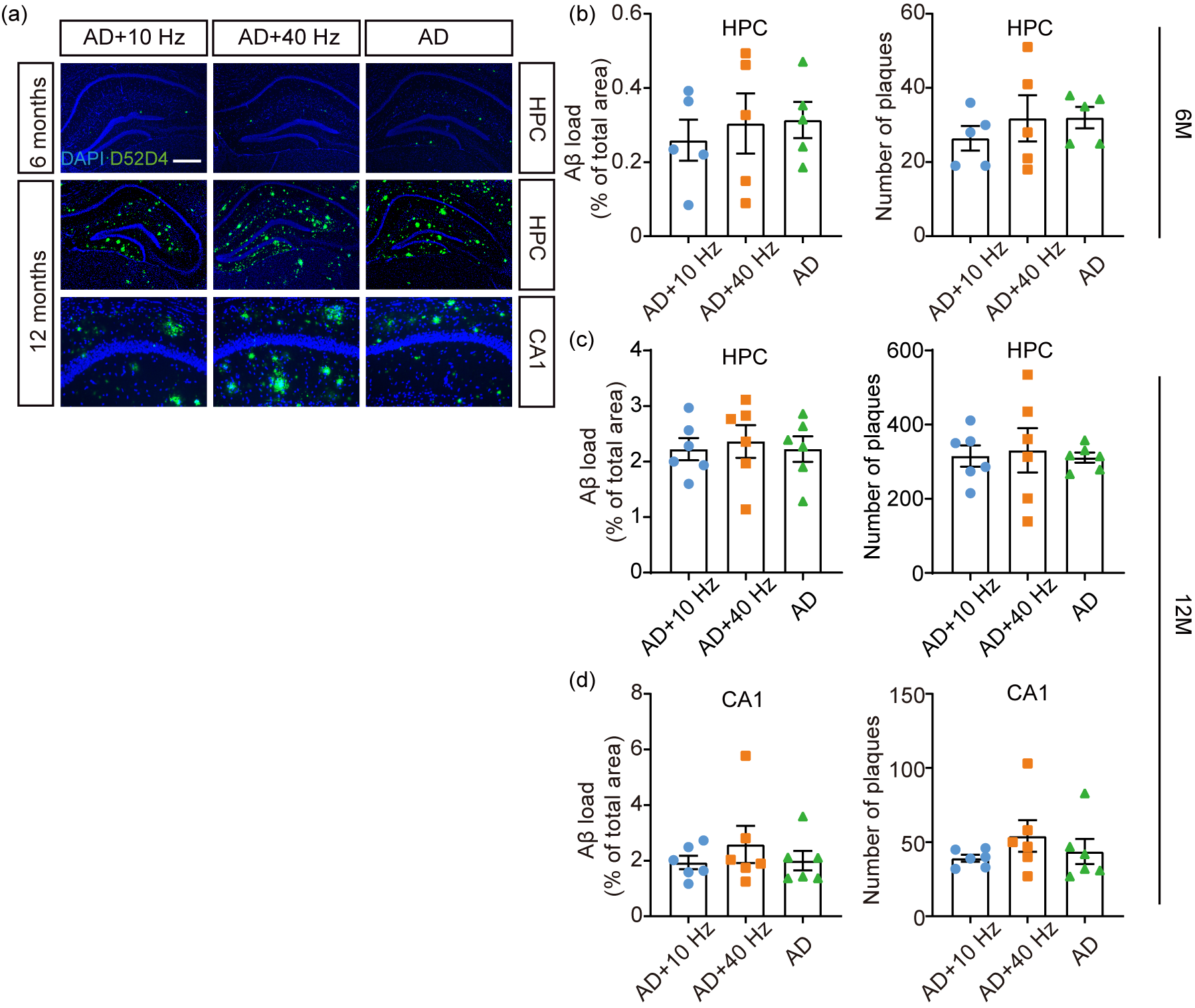


**Fig. S2.** The Aβ deposition is stained with anti-Aβ (D54D2, green) antibody in the HPC and CA1 region. (a) Immunofluorescence with anti-Aβ (D54D2, green) antibody in the HPC and CA1 region of APP/PS1 mice at 6M and 12M (scale bar, 400 μm in HPC, 100 μm in CA1 region). (b) The area and number of Aβ plaques in HPC of mice at 6M. (c), (d) The area and number of Aβ plaques in HPC (c) and CA1 region (d) of mice at 12M. Data in (b)-(d) are mean ± s.e.m., n = 5-6 per group.


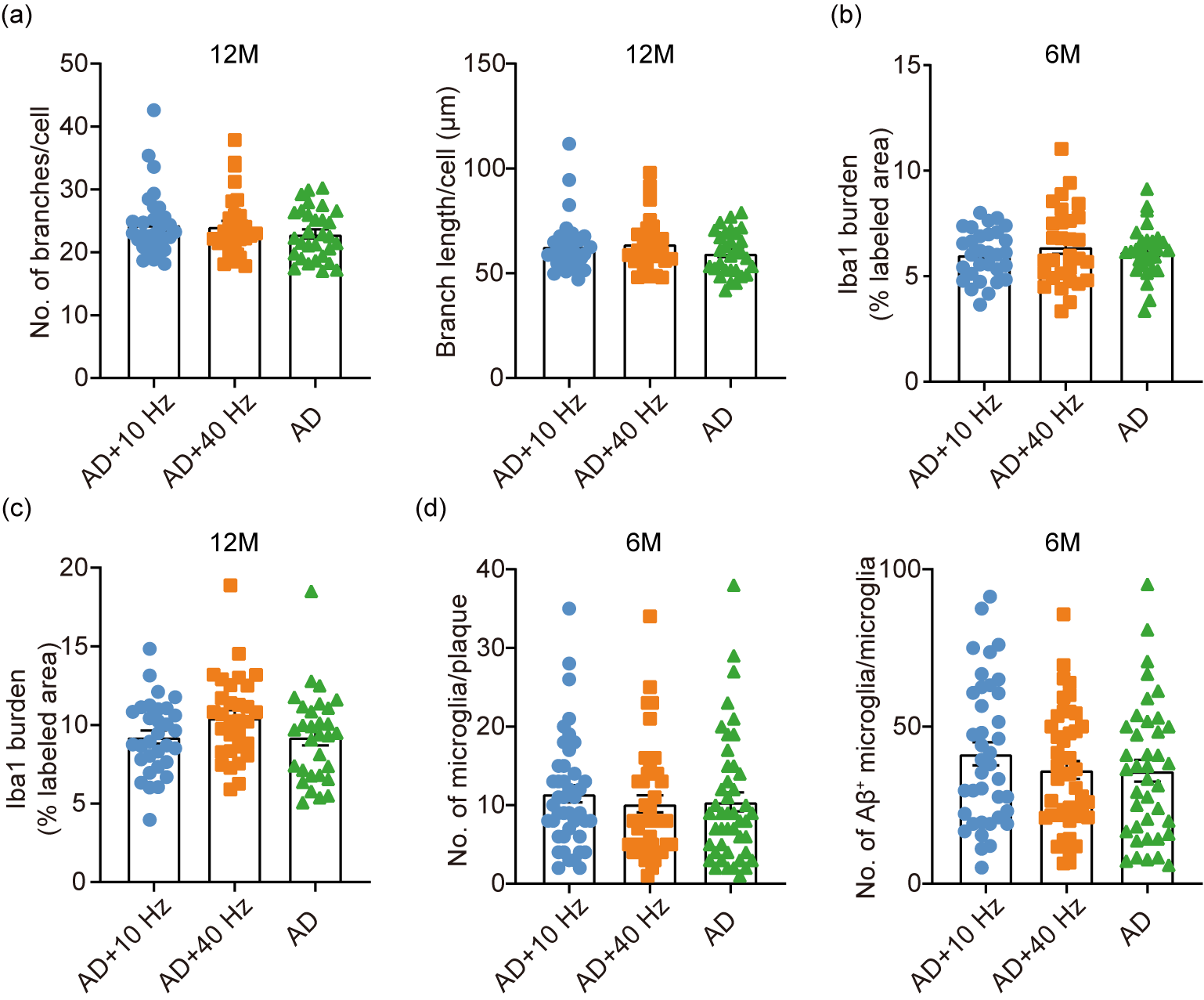


**Fig. S3.** Microglia responses to 1070-nm light in APP/PS1 mice. (a) Number and length of microglial branches per cell in the cortex of mice at 12M. (b), (c) Iba1 burden in cortex of mice at 6M (b) and 12M (c). (d) The colocalization between Aβ and microglia in mice at 6M. Data in (a)-(d) are mean ± s.e.m., n = 6 per group.


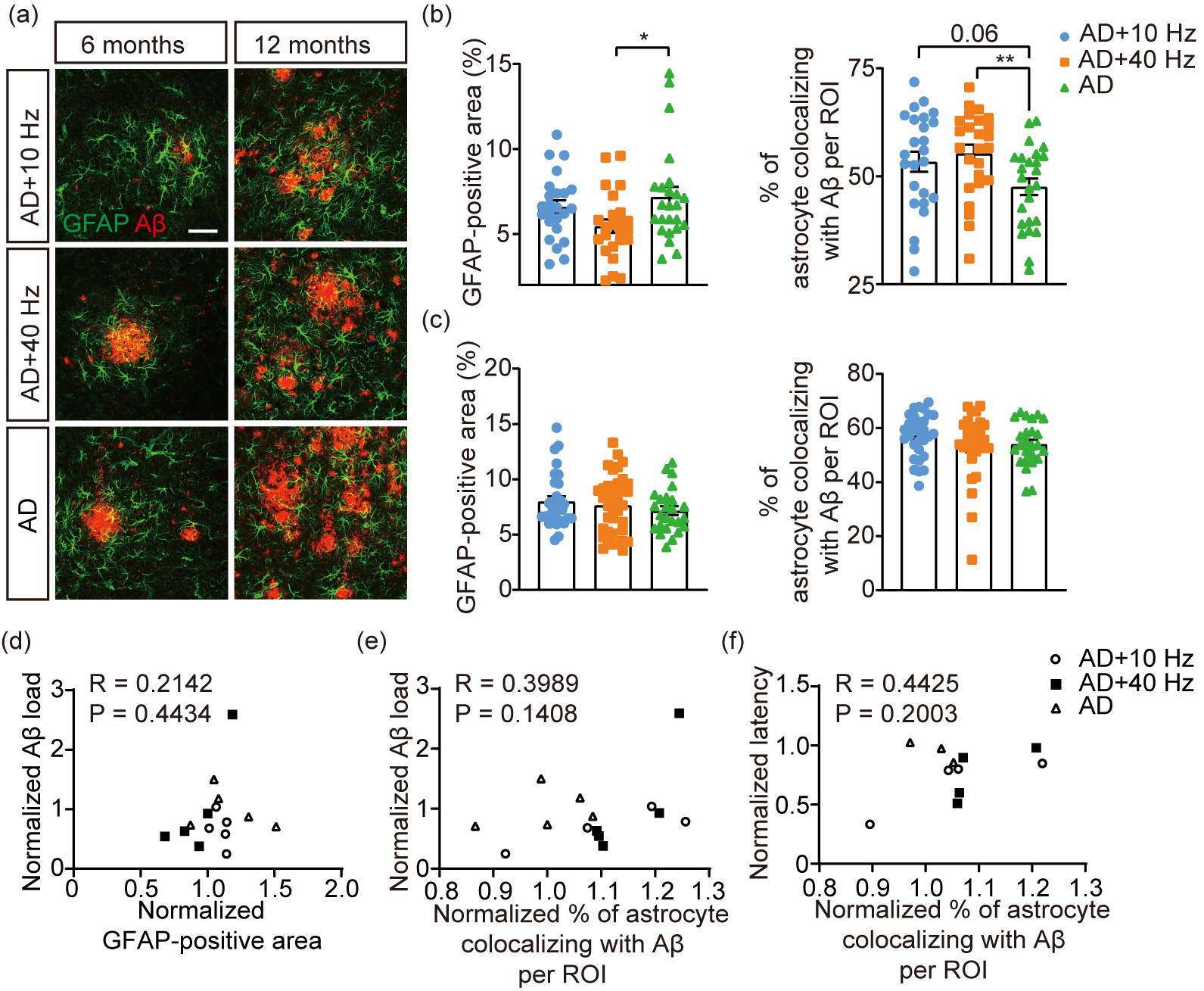


**Fig. S4.** Astrocyte responses to 1070-nm light in APP/PS1 mice. (a) Immunofluorescence with anti-GFAP (green) and anti-Aβ (D54D2, red) antibodies in the cortex of APP/PS1 mice at 6M and 12M (scale bar, 50 μm). (b), (c) Percentage of GFAP-positive area and astrocyte colocalizing with Aβ per ROI in mice at 6M (b) and 12M (c). (d) The Pearson correlation coefficient analysis between GFAP-positive area and Aβ load in mice at 6M. (e), (f) The Pearson correlation coefficient analysis between the percentage of astrocyte colocalizing with Aβ per ROI and Aβ load (e) as well as latency (f) in mice at 6M. Data in (b), (c) are mean ± s.e.m., n = 25 fields of view from 5 mice per group for (b), (c), * p < 0.05, ** p < 0.01. Data in (d)-(f) are from n = 3-5 per group.


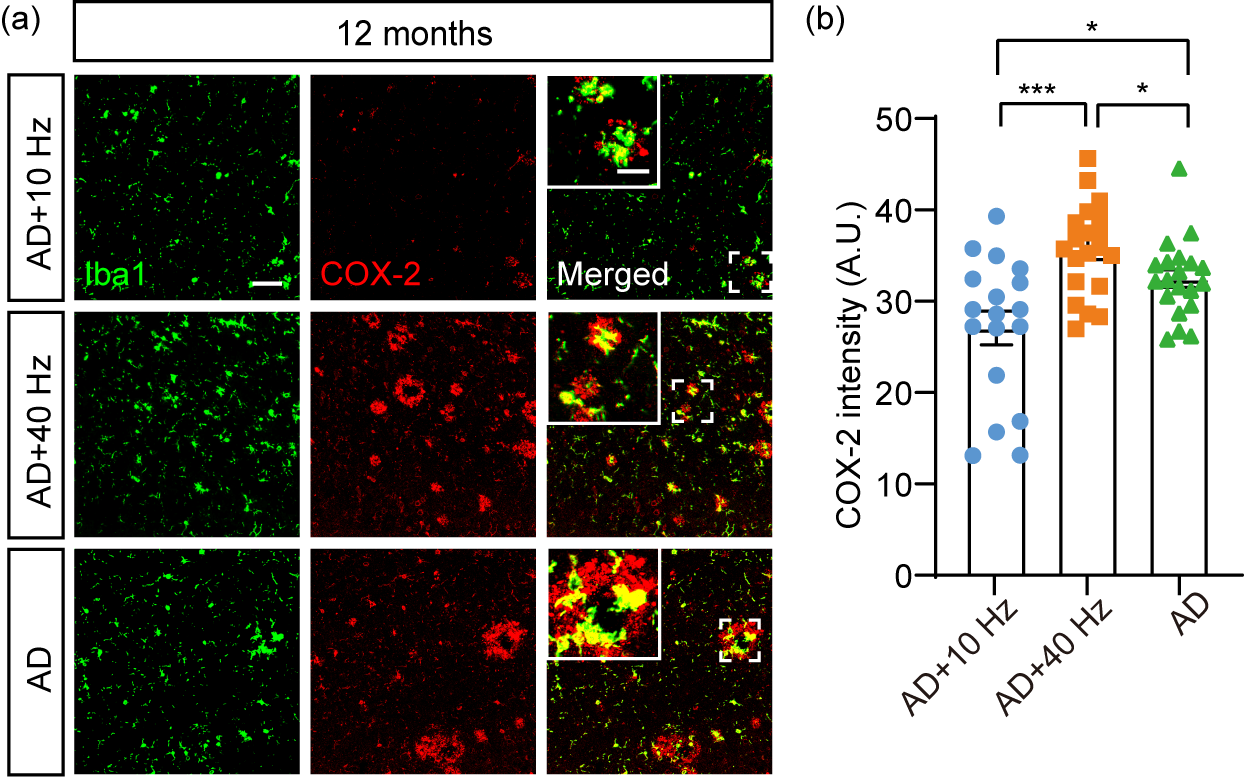


**Fig. S5.** 1070-nm light reduces M1-like microglia in the cortex of APP/PS1 mice at 12M. (a) Immunofluorescence with anti-Iba1 (green) and anti-COX-2 (red) antibodies in the cortex of APP/PS1 mice at 12M (scale bar, 50 μm). The magnified images show the typical microglia expressed with COX-2 (scale bar, 20 μm). (b) The COX-2 fluorescence intensity in mice at 12M. Data in (b) are mean ± s.e.m., n = 18-20 fields of view from 4 mice per group, * p < 0.05, ** p < 0.01, *** p < 0.001.


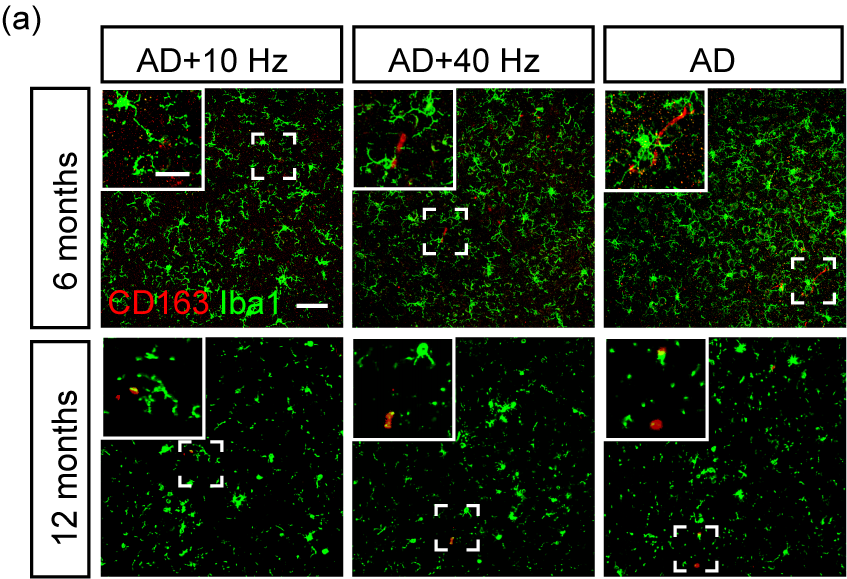


**Fig. S6.** There were not many M2-like microglia in AD mice. (a) Immunofluorescence with anti-Iba1 (green) and anti-CD163 (red) antibodies in mice at 6M and 12M (scale bar, 50 μm). The magnified images indicate the expression of CD163 (scale bar, 20 μm).
